# Thermal Analysis of 3D Electromagnetic Radiative Nanofluid Flow with Suction/Blowing: Darcy–Forchheimer Scheme

**DOI:** 10.3390/mi12111395

**Published:** 2021-11-13

**Authors:** Hammad Alotaibi, Mohamed R. Eid

**Affiliations:** 1 Department of Mathematics, College of Science, Taif University, P.O. Box 11099, Taif 21944, Saudi Arabia; 2 Department of Mathematics, Faculty of Science, New Valley University, Al-Kharga 72511, Al-Wadi Al-Gadid, Egypt; m_r_eid@yahoo.com; 3 Department of Mathematics, Faculty of Science, Northern Border University, Arar 1321, Saudi Arabia

**Keywords:** nanofluid, Darcy–Forchheimer, thermal radiation, higher-order chemical reactions, magnetic field, suction/blowing

## Abstract

This paper discusses the Darcy–Forchheimer three dimensional (3D) flow of a permeable nanofluid through a convectively heated porous extending surface under the influences of the magnetic field and nonlinear radiation. The higher-order chemical reactions with activation energy and heat source (sink) impacts are considered. We integrate the nanofluid model by using Brownian diffusion and thermophoresis. To convert PDEs (partial differential equations) into non-linear ODEs (ordinary differential equations), an effective, self-similar transformation is used. With the fourth–fifth order Runge–Kutta–Fehlberg (RKF45) approach using the shooting technique, the consequent differential system set is numerically solved. The influence of dimensionless parameters on velocity, temperature, and nanoparticle volume fraction profiles is revealed via graphs. Results of nanofluid flow and heat as well as the convective heat transport coefficient, drag force coefficient, and Nusselt and Sherwood numbers under the impact of the studied parameters are discussed and presented through graphs and tables. Numerical simulations show that the increment in activation energy and the order of the chemical reaction boosts the concentration, and the reverse happens with thermal radiation. Applications of such attractive nanofluids include plastic and rubber sheet production, oil production, metalworking processes such as hot rolling, water in reservoirs, melt spinning as a metal forming technique, elastic polymer substances, heat exchangers, emollient production, paints, catalytic reactors, and glass fiber production.

## 1. Introduction

In recent years, there has been a growing interest in studying nanofluids due to their enormous potential to enhance heat transfer. A nanofluid is a liquid suspension composed of small particles of less than 100 nm in diameter [1]. This describes an essential fluid type which can boost the thermal properties of different fluids. As a result of the inclusion of a limited number of nanoparticles according to the experimental study by Choi and Eastman [1], the effective thermal conductivity of the normal fluid is significantly enhanced. The aberrant increase of thermal conductivity and viscosity in nanofluids is investigated by [2]. The flow and heat transfer of nanofluids in moving surfaces have been studied in recent years due to their importance in industry. Considering nonlinear radiations and convective boundary conditions, Hady et al. [3] examined boundary-layer behavior on a moving surface in a nanofluid. Alotaibi et al. [4] presented a comprehensive analysis on the impact of the heat absorption and the suction on 2D Casson nanofluid flow via a non-linear stretching surface with viscous dissipation. Several works [5,6,7,8,9,10,11,12,13,14,15,16] in the field of nanofluids were conducted to demonstrate that heat transfer improves when introducing nanoparticles to the base fluid.

In many problems of practical interest, scientists and engineers have explored different methods to investigate variations in porosity, thermal distribution, local thermal equilibrium effects between the fluid and solid phases, partly complete porous structures, and anisotropic porous materials. For example, Abbas and Hayat [17] investigated thermal radiation’s effect on the two-dimensional steady flow of a viscid fluid to a non-linear, pore-saturated stretch layer. Yazdi et al. [18] probed the combined effects of the slip and chemical reaction to electrically conduct through a non-linear, porous stretch layer. Hayat et al. [19] explored the impacts of convective boundary constraints on the hydrodynamic flow of magnetic nanofluids in a porous material by considering an exponentially expanding surface. The influence of the mixed convective MHD (magnetohydrodynamic) boundary-layer of nanofluid flow on a porous exponential stretch surface was investigated by [20]. Makinde et al. [21] examined the influence of thermophoresis and radiation on the heat transfer of the varyingly viscous MHD flux through a heated flat surface on the porous material. Eid and Mahny [22] researched the flow and conversion of heat in the porous matrix of a heat source/sink by a Sisko nanofluid over an exponentially stretching layer. Eid et al. [23] investigated the effect of nanoparticles and the magnetic field on Buongiorno’s model of Carreau fluid, with the presence of both injections and thermal radiation, over the porous non-linear extending plate. In the power-law of non-Newtonian nanofluid, Eid and Mahny [24] investigated the unstable convective layer movement through the extended heat sheet. Eid et al. [25] numerically studied the convective heat and mass transportation of Carreau nanofluid in the presence of chemical reactions and an internal heat source/sink via a non-linear extended surface embedded in a porous material.

Darcy’s theory by [26] presented a more detailed review and discussed several physical applications relevant to porous media issues. The Darcy–Forchheimer equation was first introduced by Forchheimer [27] by adding the quadratic term to the transform equation. References may provide additional relevant studies on Darcy–Forchheimer flow and a variety of reports about this point in [27,28,29,30,31,32,33,34,35,36,37,38]. Although these studies focused on nanofluid flow through Darcy–Forchheimer porous materials, they were able to study specific effects, which are points of distinction for these studies, as follows: convective conditions [28], the Williamson nanofluid model [29], binary reactions [30,31], activation energy [32], second-order slip velocity [33], Ohmic heating and heat source (sink) [34], entropy production [35,36,37], magnetic Reynolds numbers [38], and electromagnetic field [39].

This article aims to develop and support the study of nanofluid flow by investigating the effect of higher-order chemical reactions with activation energy with a heat source (sink) on the 3D flow of two-phase MHD radiative nanofluid. Here, we limit our attention to discussing the Darcy–Forchheimer formula of numerical analysis for 3D two-phase permeable nanofluid flow through the porous extending sheet under thermal radiation and magnetic field impacts under convective constraint. The activation energy influence with higher-order chemical reactions in the existence of a heat source is considered because there are not only first-order chemical reactions, except in simple cases of the mathematical model, nor are they in isolation from heat sources. The governing flow equations that consist of motion, energy, and nanoparticle volume concentration formulas are facilitated by employing the similarity conversion variables. The numeric shooting technique in collaboration with the RKF45 method solves the resulting nonlinear coupled ODEs. The effect on the velocity, temperature, and concentration is computed and presented by graphs for different related parameters such as magnetic field parameters, thermal radiation, and the suction/blowing parameters. Furthermore, tables demonstrate the values of the skin friction and local Nusselt numbers.

## 2. Flow Problem Formulations

In the presence of a higher-order chemical reaction with activation energy, the steady 3D MHD incompressible nanofluid flows past the porous extending surface. The surface is assumed to be smoothly embedded in a Darcy–Forchheimer-type porous material.

**Figure 1:**
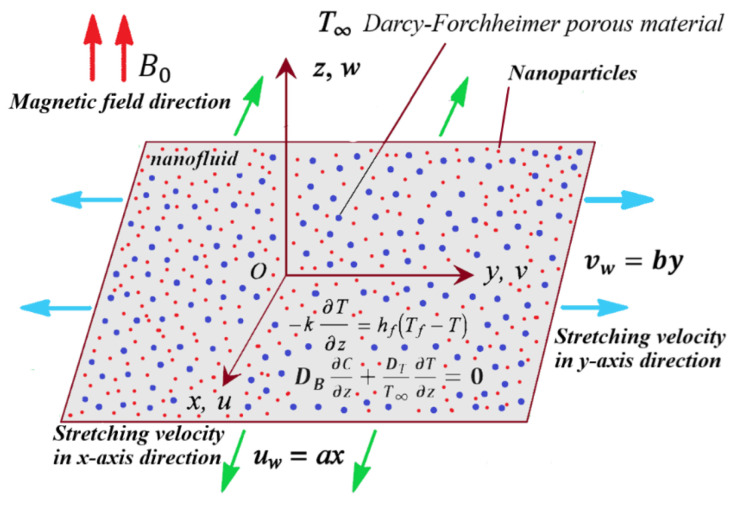
Geometry of the flow model.

Thermal radiation and the heat source are considered in the energy equation. Also, we take into account the thermophoresis and Brownian motion effects. The measured surface is extended along the x,y plane, whereas the fluid is positioned lengthways on the z-axis. It is assumed that the induced magnetic field is insignificant and the magnetic field with strength $B_{0}$ is applied perpendicular to the fluid flow. Here, we assumed that u=ax and v=by are the respective velocities along with the directions of the x-axis and y-axis with constants a and b. The geometry of the model problem of the 3D flow of the permeable nanofluid is revealed in Figure 1. The coefficient of heat transfer $h_{f}$ and temperature of the hot fluid $T_{f}$ below the surface temperature are governed by a convection heating operation.

With the above suggestions and boundary layer approximations, the momentum boundary-layer formulas of two-phase nanofluid flow are as follows [28,29]:

(1) $$\frac{\partial u}{\partial x}+\frac{\partial v}{\partial y}+\frac{\partial w}{\partial z}=0$$

(2) $$u\frac{\partial u}{\partial x}+v\frac{\partial u}{\partial y}+w\frac{\partial u}{\partial z}=\nu_{f}\frac{\partial^{2}u}{\partial z^{2}}-\frac{\sigma B_{0}^{2}}{\rho_{f}}\;u-\frac{\nu_{f}}{K}u-Fu^{2},\;$$

(3) $$u\frac{\partial v}{\partial x}+v\frac{\partial v}{\partial y}+w\frac{\partial v}{\partial z}=\nu_{f}\frac{\partial^{2}v}{\partial z^{2}}-\frac{\sigma B_{0}^{2}}{\rho_{f}}\;v-\frac{\nu_{f}}{K}v-Fv^{2},$$

(4) $$u\frac{\partial T}{\partial x}+v\frac{\partial T}{\partial y}+w\frac{\partial T}{\partial z}=\alpha^{*}\frac{\partial^{2}T}{\partial z^{2}}+\frac{{\left(\rho c\right)}_{p}}{{\left(\rho c\right)}_{f}}\left[D_{B}\left(\frac{\partial T}{\partial z}\frac{\partial C}{\partial z}\right)+\frac{D_{T}}{T_{\infty }}{\left(\frac{\partial T}{\partial z}\right)}^{2}\right]+\frac{Q}{{\left(\rho c\right)}_{f}}\left(T-T_{\infty }\right)-\frac{1}{{\left(\rho c\right)}_{f}}\frac{\partial q_{r}}{\partial z},$$

(5) $$u\frac{\partial C}{\partial x}+v\frac{\partial C}{\partial y}+w\frac{\partial C}{\partial z}=D_{B}\left(\frac{\partial^{2}C}{\partial z^{2}}\right)+\frac{D_{T}}{T_{\infty }}\left(\frac{\partial^{2}T}{\partial z^{2}}\right)-R{\left(\frac{T}{T_{\infty }}\right)}^{m}\exp\left(-\frac{E_{a}}{\kappa T}\right){\left(C-C_{\infty }\right)}^{n}.$$

These formulas are subject to the following boundary conditions:

(6) $$\begin{array}{c} u=U_{w}=ax,\;v=V_{w}=by,\;w=0,-k\frac{\partial T}{\partial z}=h_{f}\left(T-T_{\infty }\right),\;\;D_{B}\frac{\partial C}{\partial z}+\frac{D_{T}}{T_{\infty }}\frac{\partial T}{\partial z}=0\;\;at\;z=0,\\ u\to 0,v\to 0,\;\;T\to T_{\infty },\;\;C\to C_{\infty }\;\;as\;\;z\to \infty .\;\;\;\; \end{array}\}$$

The convective boundary constraint here, also known as the Newton boundary constraint, is derived from the surface energy balance and relates to the occurrence of convective heating (or cooling) at the surface in heat transport issues. Also, at the boundary, the zero-mass fluxing constraint is considered. Here u,v, and w are the flow velocities in the x-, y-, and z-directions, $F=\frac{C_{b}}{x\sqrt{K}}$ is the coefficient of the non-uniform inertia of the porous material,$\;C_{b}$ is the coefficient of drag, K is the porous material permeability, k is thermal conductivity, $\mu_{f}$ is the dynamic viscosity, $\nu_{f}=\frac{\mu_{f}}{\rho_{f}}$ is the kinematic viscosity, $\rho_{f}$ is the density, a and  b are the material constants, σ is the electrical conductivity, $\alpha^{*}=\frac{k}{{\left(\rho c\right)}_{f}}$ is the thermal diffusivity, ${\left(\rho c\right)}_{f}$ and ${\left(\rho c\right)}_{p}$ are the heat capacities of the fluid and nanoparticles, Q is the heat generation/absorption coefficient, T and C stand for the temperature and concentration, $T_{\infty }$ and $C_{\infty }$ are the ambient temperature and concentration, $D_{B}$ and $D_{T}$ are the Brownian motion and thermophoresis, $R{\left(\frac{T}{T_{\infty }}\right)}^{m}\exp\left(-\frac{E_{a}}{\kappa T}\right){\left(C-C_{\infty }\right)}^{n}$ is the modified Arrhenius equation in which R is the reaction rate, $E_{a}$ is the activation energy, κ is the Boltzmann constant, m is the fitted rate constant, and n is the order of a chemical reaction. The Roseland approximation is used to give the radiative heat flux expression as follows [4]:

(7) $$q_{r}=-\frac{4\sigma^{*}}{3k^{*}}\frac{\partial T^{4}}{\partial z},$$

where $k^{*}$ and $\sigma^{*}$ are the coefficient of mean absorption and the Stefan–Boltzmann constant, respectively. Given the temperature variance within the flow, the issue is such that $T^{4}$ can be expanded in a Taylor series of $T_{\infty }$ and neglect higher-order expressions. The subsequent results are approximated as

(8) $$T^{4}\approx 4T_{\infty }^{3}T-3T_{\infty }^{4}$$

By using Equations (7) and (8), we obtain the following form:

(9) $$\frac{\partial q_{r}}{\partial z}=-\frac{16\sigma^{*}T_{\infty }^{3}}{3k^{*}}\frac{\partial^{2}T}{\partial z^{2}}$$

Consequently, through Equation (9), the heat Equation (4) converts to

(10) $$u\frac{\partial T}{\partial x}+v\frac{\partial T}{\partial y}+w\frac{\partial T}{\partial z}=\left[\alpha^{*}+\frac{16\sigma^{*}T_{\infty }^{3}}{3k^{*}{\left(\rho c_{p}\right)}_{f}}\right]\frac{\partial^{2}T}{\partial z^{2}}+\frac{{\left(\rho c\right)}_{p}}{{\left(\rho c\right)}_{f}}\left[D_{B}\left(\frac{\partial T}{\partial z}\frac{\partial C}{\partial z}\right)+\frac{D_{T}}{T_{\infty }}{\left(\frac{\partial T}{\partial z}\right)}^{2}\right]+\frac{Q}{{\left(\rho c\right)}_{f}}\left(T-T_{\infty }\right)$$

Introducing similarity transformations [32,36], we are given

(11) $$\begin{array}{c} u={axf}^{\prime }\left(\zeta \right),\;v={ayg}^{\prime }\left(\zeta \right),\;w=-\sqrt{a\nu_{f}}\;\left[f\left(\zeta \right)+\;g\left(\zeta \right)\right],\\ \;\theta \left(\zeta \right)=\frac{T-T_{\infty }}{T_{f}-T_{\infty }},\;\phi \left(\zeta \right)=\frac{C-C_{\infty }}{C_{\infty }},\;\zeta =\sqrt{\frac{a}{\nu_{f}}}z.\; \end{array}\}$$

Equation (1) is now satisfied and Equations (2), (3), (5), (6), and (10) reduce to the following non-dimensional form:

The non-linear system:

(12) $$f^{\prime \prime \prime }+\left(f+\;g\right)f^{\prime \prime }-\left(1+F_{r}\right)\;{f^{\prime }}^{2}-\left(M^{2}+\lambda \right)f^{\prime }=0,$$

(13) $$g^{\prime \prime \prime }+\left(f+\;g\right)\;g^{\prime \prime }-\left(1+F_{r}\right){\;g^{\prime }}^{2}-\left(M^{2}+\lambda \right)\;g^{\prime }=0,$$

(14) $$\left(1+\frac{4}{3}R_{d}\right)\theta^{\prime \prime }+Pr\left[\left(f+g\right)\theta^{\prime }+N_{b}\theta^{\prime }\phi^{\prime }+N_{t}{\theta^{\prime }}^{2}+S\theta \right]=0,$$

(15) $$\phi^{\prime \prime }+\frac{N_{t}}{N_{b}}\theta^{\prime \prime }+Sc\left[\left(f+g\right)\phi^{\prime }-\gamma {\left(1+\delta \theta \right)}^{m}\exp\left(-\frac{E}{1+\delta \theta }\right)\phi^{n}\right]=0.$$

The transformed boundary conditions are

(16) $$\begin{array}{c} f\left(0\right)=g\left(0\right)=0,\;f^{\prime }\left(0\right)=1,\;g^{\prime }\left(0\right)=\alpha ,\;\theta^{\prime }\left(0\right)=-Bi\left[1-\theta \left(0\right)\right],N_{b}\theta^{\prime }\left(0\right)+N_{t}\phi^{\prime }\left(0\right)=0,\\ f^{\prime }\left(\infty \right)\to 0,g^{\prime }\left(\infty \right)\to 0,\;\;\theta \;\left(\infty \right)\to 0,\;\;\phi \left(\infty \right)\to 0,\;\;\;\;\;\;\;\;\;\;\;\;\;\;\;\;\;\;\;\; \end{array}\},$$

where the prime here is referring to the similarity variable ζ, $F_{r}$ is the Forchheimer number, M is a magnetic parameter, λ is the permeability parameter, $\;R_{d}$ is the radiation parameter,  Pr is the Prandtl number, $N_{b}$ is the Brownian motion, $N_{t}$ is the thermophoresis diffusion, *S* is the heat source/sink parameter, Sc is Sthe chmidt number, γ represents the chemical reaction parameter, δ is the temperature relative parameter, E is the activation energy, α is a material parameter, and Bi is the Biot number. These parameters are characterized by

(17) $$\begin{array}{c} F_{r}=\frac{C_{b}}{\sqrt{K}},M^{2}=\frac{\sigma B_{0}^{2}}{a\rho_{f}},\;\lambda =\frac{\nu_{f}}{aK},\;R_{d}=\frac{4\sigma^{*}T_{\infty }^{3}}{k^{*}k\;},Pr=\frac{\nu_{f}}{\alpha },\\ N_{b}=D_{B}\frac{{\left(\rho c\right)}_{p}}{{\left(\rho c\right)}_{f}}\frac{C_{\infty }}{\nu_{f}{}_{\;}}\;,\;N_{t}=\frac{D_{T}}{T_{\infty }}\;\frac{{\left(\rho c\right)}_{p}}{{\left(\rho c\right)}_{f}}\frac{\left(T_{f}-T_{\infty }\right)}{\nu_{f}},\;S=\frac{Q}{a{\left(\rho c\right)}_{f}},Sc=\frac{\nu_{f}}{{\;}_{\;}D_{B}}\\ \gamma =\frac{RC_{\infty }^{n-1}}{a},\;\;\;\delta =\frac{\left(T_{f}-T_{\infty }\right)}{T_{\infty }},\;E=\frac{E_{a}}{\kappa T_{\infty }},Bi=\frac{h_{f}}{k}\sqrt{\frac{\nu_{f}}{a}},\alpha =\frac{b}{a}.\;\;\;\;\; \end{array}\}$$

The quantities of physical interest such as the non-dimensional coefficient of skin friction, the couple stress, and the Nusselt number are defined as follows [40]:

(18) $$\begin{array}{c} C_{f}=\frac{\tau_{w}}{\rho_{f}U_{w}^{2}},\;\tau_{w}=\mu_{f}{\left(\frac{\partial u}{\partial z}\right)}_{z=0},\\ C_{f}=\frac{\tau_{w}}{\rho_{f}V_{w}^{2}},\;\tau_{w}=\mu_{f}{\left(\frac{\partial v}{\partial z}\right)}_{z=0},\\ Nu=\frac{xq_{w}}{k_{f}\left(T_{w}-T_{\infty }\right)},\;\;\;\;\;\;\;\;\;\;\;\;\;\;\;\;\;\;\;\;\;\; \end{array}\}$$

where $\tau_{w}$ and $q_{w}$ signify shear stress and heat fluxing. The dimensionless form of Equation (18) after utilizing the similarity conversions in Equation (11) becomes

(19) $$\begin{array}{c} C_{fx}Re_{x}^{1/2}=-f^{\prime \prime }\left(0\right),\;\\ C_{fy}Re_{x}^{1/2}=\alpha^{-3/2}g^{\prime \prime }\left(0\right),\;\\ Nu_{x}Re_{x}^{-1/2}=-\theta^{\prime }\left(0\right), \end{array}\},$$

where $Re_{x}=\frac{U_{w}x}{\upsilon }$ and $Re_{y}=\frac{V_{w}y}{\upsilon }$ describe the local Reynolds numbers. 

## 3. The RFK45 Technique

RFK45 [25] is utilized to solve the problem in the form $\frac{dy}{dx}=f\left(x,y\right),\;y\left(x_{i}\right)=y_{i}$ for i=0, 1,…,N. The solution accuracy of the above Equation is determined by applying the appropriate step size. Two solutions are assessed and compared. If the majority of the values agree, the approximation is valid. The step size is adjusted if the approximations are not near enough to each other to achieve the specified precision. If the values match more than the significant digits, a step-size increase is produced. The following six steps are wanted in each step:

(20) $$\begin{array}{c} k_{1}=hf\left(x_{i},y_{i}\right),\\ k_{2}=hf\left(x_{i}+\frac{1}{4}h,y_{i}+\frac{1}{4}k_{1}\right),\\ k_{3}=hf\left(x_{i}+\frac{3}{8}h,y_{i}+\frac{3}{32}k_{1}+\frac{9}{32}k_{2}\right),\\ k_{4}=hf\left(x_{i}+\frac{12}{13}h,y_{i}+\frac{1932}{2197}k_{1}-\frac{7200}{2197}k_{2}+\frac{7296}{2197}k_{3}\right),\\ k_{5}=hf\left(x_{i}+h,y_{i}+\frac{439}{216}k_{1}-8k_{2}+\frac{3680}{513}k_{3}-\frac{845}{4104}k_{4}\right),\\ k_{6}=hf\left(x_{i}+\frac{1}{2}h,y_{i}-\frac{8}{27}k_{1}+2k_{2}-\frac{3544}{2565}k_{3}+\frac{1859}{4104}k_{4}-\frac{11}{40}k_{5}\right). \end{array}\},$$

The fourth-order estimate is

(21) $$y_{i}=x_{i}+\frac{25}{216}k_{1}+\frac{1408}{2565}k_{3}+\frac{2197}{4101}k_{4}-\frac{1}{5}k_{5},$$

The fifth-order estimate is specified by

(22) $$z_{i+1}=y_{i}+\frac{16}{135}k_{1}+\frac{6656}{12825}k_{3}+\frac{28561}{56430}k_{4}-\frac{9}{50}k_{5}+\frac{2}{55}k_{6}.$$

Eventually, the optimal step-size δ is attained by multiplying h with a scalar δ, where δ is itemized by

(23) $$\delta =0.84{\left(\frac{\varepsilon h}{2\left|z_{i+1}-y_{i+1}\right|}\right)}^{0.25},\;$$

where ε is the error tolerance. The boundary value problem in (12)–(16) converts to an initial value problem as follows:

(24) $${ℋ}_{1}=f,\;{ℋ}_{2}=f^{\prime },\;{ℋ}_{3}=f^{\prime \prime },\;{ℋ}_{4}=g,\;{ℋ}_{5}=\;g^{\prime },\;{ℋ}_{6}=\;g^{\prime \prime },\;{ℋ}_{7}=\theta ,\;{ℋ}_{8}=\theta^{\prime },\;{ℋ}_{9}=\phi ,\;{ℋ}_{10}=\phi^{\prime },$$

By substituting (24) into (12)–(16), the result is the reduced first-order scheme of Equations:

(25) $${{ℋ}_{3}}^{\prime }+\left({ℋ}_{1}+{ℋ}_{4}\right){ℋ}_{3}-\left(1+F_{r}\right)\;{{ℋ}_{2}}^{2}-\left(M^{2}+\lambda \right){ℋ}_{2}=0,$$

(26) $${{ℋ}_{6}}^{\prime }+\left({ℋ}_{1}+{ℋ}_{4}\right){ℋ}_{6}-\left(1+F_{r}\right)\;{{ℋ}_{5}}^{2}-\left(M^{2}+\lambda \right){ℋ}_{5}=0,$$

(27) $$\left(1+\frac{4}{3}R_{d}\right){{ℋ}_{8}}^{\prime }+Pr\left[\left({ℋ}_{1}+{ℋ}_{4}\right){ℋ}_{8}+N_{b}{ℋ}_{8}{ℋ}_{10}+N_{t}{{ℋ}_{8}}^{2}+S{ℋ}_{7}\right]=0,$$

(28) $${{ℋ}_{10}}^{\prime }+\frac{N_{t}}{N_{b}}{{ℋ}_{8}}^{\prime }+Sc\left[\left({ℋ}_{1}+{ℋ}_{4}\right){ℋ}_{10}-\gamma {\left(1+\delta {ℋ}_{7}\right)}^{m}\exp\left(-\frac{E}{1+\delta {ℋ}_{7}}\right){{ℋ}_{9}}^{n}\;\right]=0.$$

The transformed boundary conditions are

(29) $$\begin{array}{c} {ℋ}_{1}\left(0\right)={ℋ}_{4}\left(0\right)=0,{ℋ}_{2}\left(0\right)=1,\;{ℋ}_{5}\left(0\right)=\alpha ,\\ {ℋ}_{8}\left(0\right)=-Bi\left[1-{ℋ}_{7}\left(0\right)\right],N_{b}{ℋ}_{8}\left(0\right)+N_{t}{ℋ}_{10}\left(0\right)=0,\\ {ℋ}_{2}\left(\infty \right)\to 0,{ℋ}_{5}\left(\infty \right)\to 0,\;{ℋ}_{7}\left(\infty \right)\to 0,\;{ℋ}_{9}\left(\infty \right)\to 0. \end{array}\}$$

The criterion of convergence is determined to be at least $10^{-6}$.

## 4. Results and Discussion

**Figure 2:**
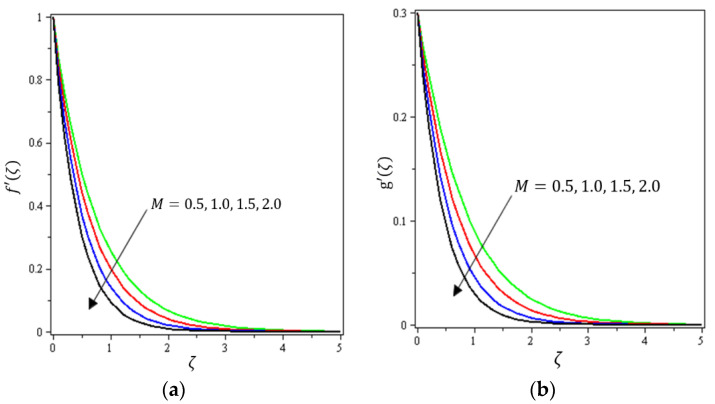
Influence of magnetic field M on velocities (**a**) $f{}^{\prime }\left(\zeta \right)$ and (**b**) *g*${}^{\prime }\left(\zeta \right)$.

**Figure 3:**
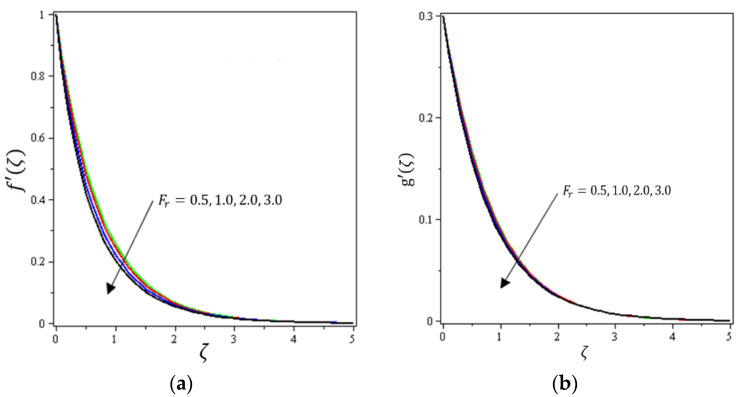
Influence of Darcy–Forchheimer number $F_{r}$ on velocities (**a**) $f{}^{\prime }\left(\zeta \right)$ and (**b**) *g*${}^{\prime }\left(\zeta \right)$.

**Figure 4:**
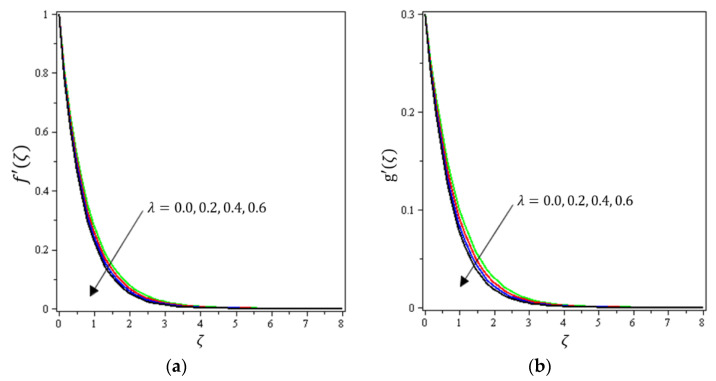
Influence of permeability parameter λ on velocities (**a**) $f{}^{\prime }\left(\zeta \right)$ and (**b**) *g*${}^{\prime }\left(\zeta \right)$.

**Figure 5:**
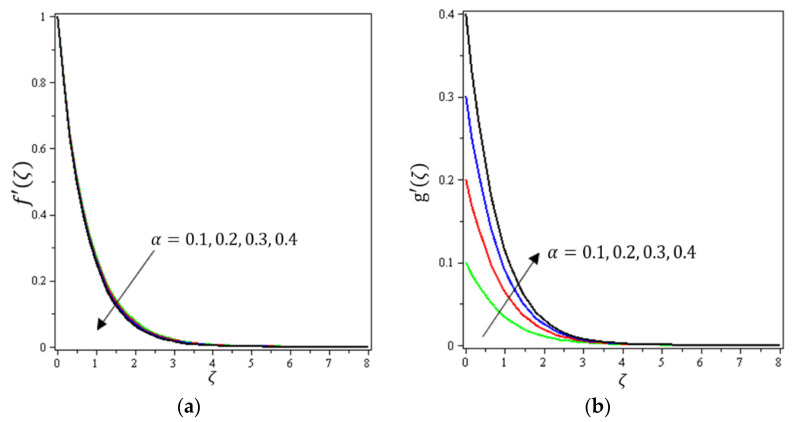
Influence of material parameter α on velocities (**a**) $f{}^{\prime }\left(\zeta \right)$ and (**b**) *g*${}^{\prime }\left(\zeta \right)$.

**Figure 6:**
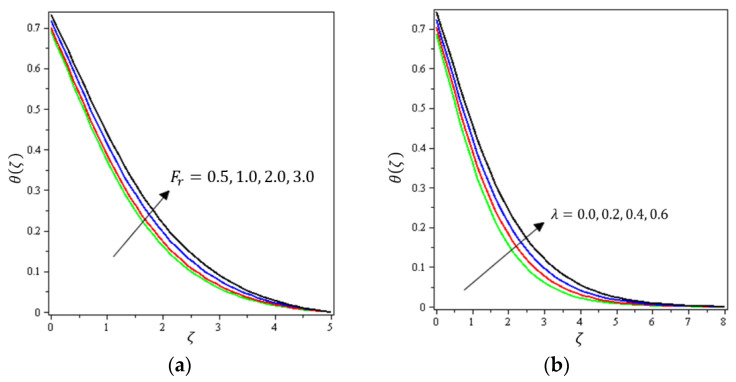
Influence of (**a**) Darcy–Forchheimer $F_{r}$ and (**b**) Prandtl number Pr on θ(ζ).

**Figure 7:**
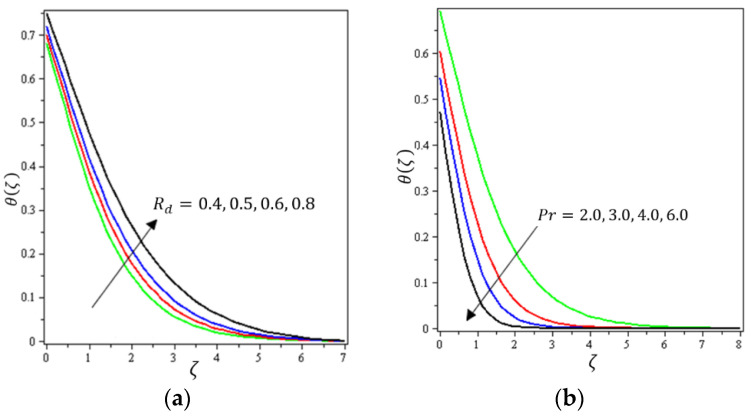
Influence of (**a**) radiative flow $R_{d}$ and (**b**) heat-generating S on θ(ζ).

**Figure 8:**
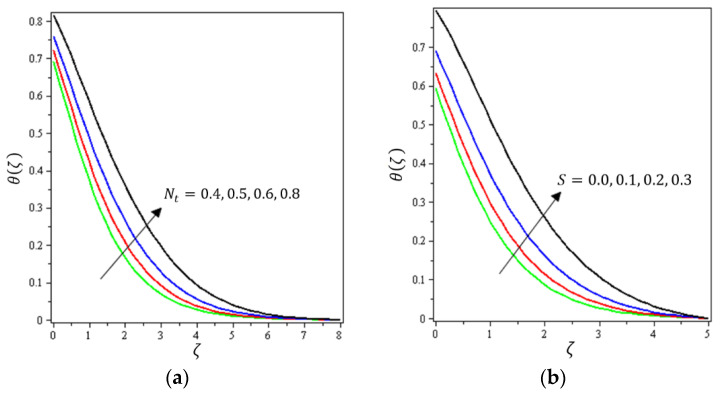
Influence of (**a**) thermophoretic diffusion $N_{t}$ and (**b**) permeability parameter λ on θ(ζ).

**Figure 9:**
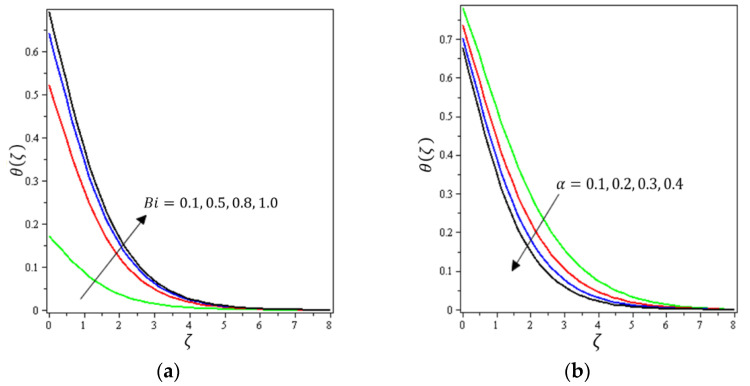
Influence of (**a**) Biot number Bi and (**b**) material parameter α on θ(ζ).

**Figure 10:**
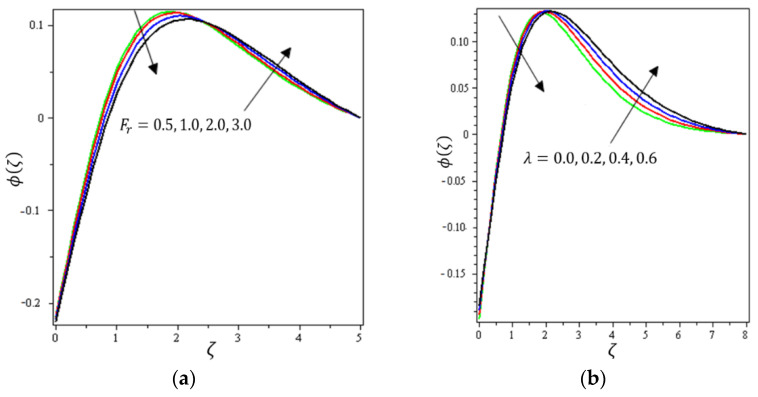
Influence of (**a**) Darcy–Forchheimer $F_{r}$ and (**b**) permeability parameter λ on ϕ(ζ).

**Figure 11:**
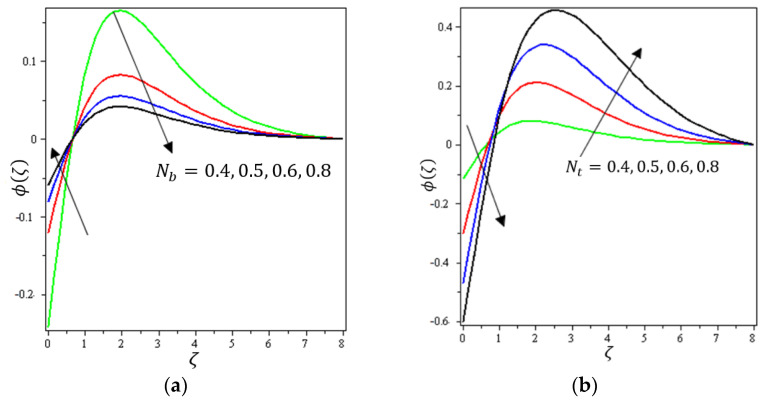
Influence of (**a**) Brownian movement $N_{b}$ and (**b**) thermophoretic diffusion $N_{t}$ on ϕ(ζ).

**Figure 12:**
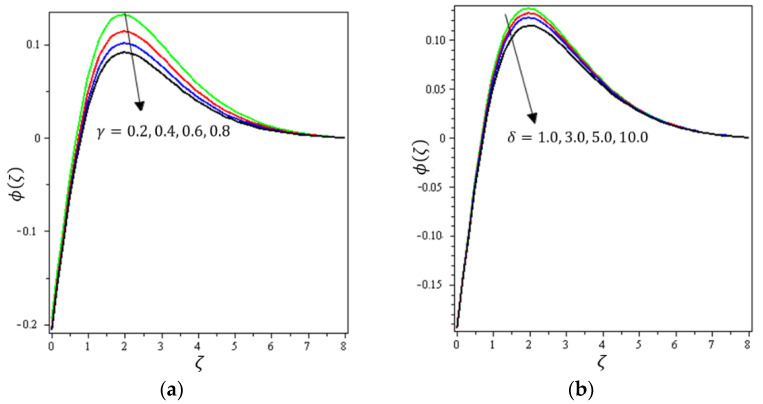
Influence of (**a**) chemical reactive γ and (**b**) temperature ratio δ on ϕ(ζ).

**Figure 13:**
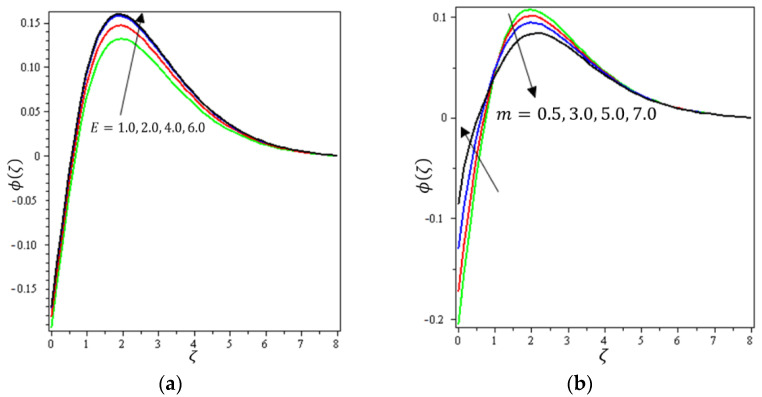
Influence of (**a**) activation energy E and (**b**) fitted rate m on ϕ(ζ).

**Figure 14:**
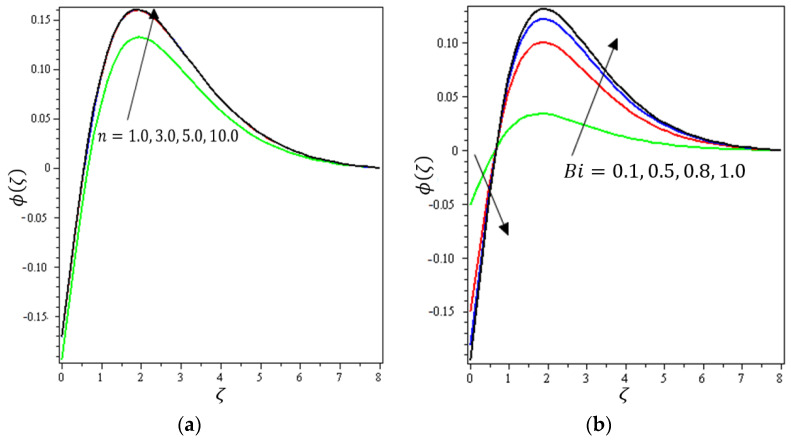
Influence of (**a**) chemical order n and (**b**) Biot number Bi on ϕ(ζ).

This section aims to discuss and present the effect of different physical parameters. Figure 2, Figure 3, Figure 4, Figure 5, Figure 6, Figure 7, Figure 8, Figure 9, Figure 10, Figure 11, Figure 12, Figure 13 and Figure 14 present the quantities of physical interest such as the velocity profile $f^{\prime }\left(\zeta \right)$ in the x-direction, the velocity profile $g^{\prime }\left(\zeta \right)$ in the y-direction, the temperature profile  θ(ζ), and the concentration profile ϕ(ζ), considering the effect of physical parameters such as the Forchheimer number $\;F_{r}$, the magnetic parameter M, the permeability parameter λ, the radiation parameter $R_{d}$, the Prandtl number  Pr, the Brownian motion parameter $N_{b}$, the thermophoresis parameter $N_{t}$, the heat generation/absorption parameter S, the Schmidt number  Sc, the chemical reaction parameter γ, the temperature relative parameter δ, the activation energy E, the fitted rate constant m, the order of the chemical reaction n, the ratio parameter α, and the Biot number Bi. For the results, we considered, $M=F_{r}=R_{d}=N_{b}=N_{t}=m=0.5$, Pr=2, E=Bi=n=Sc=δ=1, S=γ=λ=0.2, and  α=0.3. 

Figure 2, Figure 3a, Figure 4a and Figure 5a depict the effects of M, $F_{r}$, λ, and α on $f^{\prime }\left(\zeta \right)$, respectively. It can be noted from these figures that the flow velocity decreases with rising values of the magnetic field, Forchheimer number, permeability parameter, and material parameter. The reason is the potentiality of the Lorentz force, which takes place due to the magnetic field, where the corresponding boundary-layer appears extra thick and the movement of the fluid cannot be easy. Figure 2b, Figure 3b, Figure 4b and Figure 5b illustrate the influences of M, $F_{r}$, λ, and α on $g^{\prime }\left(\zeta \right)$, respectively. It can be observed from these figures that the velocity reduces with increasing values of the magnetic field, Forchheimer number, permeability parameter, and ratio parameter. This indicates that the magnetic field reduces collisions between the nanofluid particles, which reduces the velocities in both the horizontal and vertical directions. Figure 6a shows the impact of $F_{r}$ on θ(ζ). It was found that an increment in Forchheimer’s number causes temperature improvement and boundary layer thickness. Figure 6b exhibits the effect of λ on $\theta \left(\zeta_{\;}\right)$. It can be seen that the presence of the permeability parameter augments the resistance contra the flow of the fluid, which results in a stronger temperature profile. Figure 7a displays the impact of $R_{d}$ on θ(ζ). It can be noted that $\theta \left(\zeta_{\;}\right)$ enhances with increasing values of the radiation parameter. Figure 7b presents the effect of Pr on  θ(ζ). It can be interpreted that θ(ζ) dwindles with the growing values of the Prandtl number. Physically, the thermal diffusivity deteriorates for large Pr. The Prandtl number influences the thermal and momentum boundary layers. When Pr is small, the temperature diffuses quickly relative to the velocity. Figure 8a shows the influence of $N_{t}$ on θ(ζ). It can be noted that θ(ζ) enhances with the higher values of the thermophoresis parameter. Figure 8b shows the effect of S on  θ(ζ); it can be seen that θ(ζ) rises with the enhanced values of the heat source/sink parameter. This means that the nanofluid has less heat transfer, which allows it to maintain its temperature for a longer period with the presence of a heat source in the beginning. Figure 9a plots the effect of Bi on $\theta \left(\zeta_{\;}\right)$. It can be seen that θ(ζ) increases with the higher values of the Biot number. The thickness of the thermal boundary also increases with a rise in the Biot number. Figure 9b describes the influence of α on $\theta \left(\zeta_{\;}\right)$. It can be noted that $\theta \left(\zeta_{\;}\right)$ declines with higher values of the ratio parameter.

Variations of the $F_{r}$, λ,$\;R_{d}$, Pr,$\;N_{b}$, $N_{t}$, S, Sc, γ, δ, E, m, n, and Bi parameters on ϕ(ζ) are presented in Figure 10, Figure 11, Figure 12, Figure 13 and Figure 14. Figure 10a presents the effect of $F_{r}$ on $\phi \left(\zeta_{\;}\right)$. It shows that $\phi \left(\zeta_{\;}\right)$ declines with the higher values of $F_{r}$ up to a certain distance from the stretching sheet (about ζ=2.5). After this point, an increase in the values of $F_{r}$ produces an increase in the volume concentration distribution. Figure 10b indicates the effect of λ on ϕ(ζ). It is obvious that an increase in the values of the permeability parameter reduces ϕ(ζ) up to a certain distance from the stretching sheet (about ζ=2.5); after this point, an increase in the values of λ leads to an increment in the concentration profile. Figure 11a shows the impact of $N_{b}$ on ϕ(ζ). It was found that ϕ(ζ) increases with the higher values of $N_{b}$ up to a certain distance from the stretching sheet (about ζ=1); after this point, an increase in the value of $N_{b}$ leads to a decline in the concentration profile. Brownian motion force essentially tends to move particles in opposite directions. Therefore, the higher the Brownian force, the weaker the nanoparticle concentration. Figure 11b elaborates on the impact of $N_{t}$ on ϕ(ζ). It is clear that ϕ(ζ) diminishes with higher amounts of the thermophoresis parameter up to a certain distance from the stretching sheet (about ζ=1); after this point, the opposite happens, with an increase in $N_{b}$ leading to an increment in the concentration profile. 

Figure 12a indicates the effect of γ on ϕ(ζ). It can be noticed that ϕ(ζ) decreases with increasing values of the chemical reaction parameter. For that, the reaction rate can be calculated by how rapidly the levels of concentration of a nanofluid decline. Figure 12b shows the impact of  δ on  ϕ(ζ); it can be seen that ϕ(ζ) declines with higher values of the temperature relative parameter. Figure 13a describes the influence of E on ϕ(ζ); it is clear that  ϕ(ζ) increases with increasing values of the activation energy. The Arrhenius functions physically weaken with the increase of the activation energy value, which contributes to an increase of the concentrations profile in the obstetrical chemical reaction. Figure 13b demonstrates the effect of m on  ϕ(ζ); it was found that ϕ(ζ) increases with an increase of the fitted rate constant until arriving at point ζ=1.5; after that, an increase in m leads to a decrement in  ϕ(ζ). Figure 14a depicts the impact of  n on ϕ(ζ); it can be interpreted that ϕ(ζ) increases with an increase in values of n. A certain number of molecules with energies equal to or larger than the activation energy must exist for a chemical reaction to occur. As the concentration increases, the number of molecules with the minimum energy available is increased and the reaction rate increases. Figure 14b shows the effect of Bi on ϕ(ζ); it can be seen that ϕ(ζ) declines with higher values of the Biot number until arriving at point ζ=1. After that, an increase in Bi strengthens the concentration profile. It can be noted that the concentration boundary thickness is also boosted with an increase in Bi.

Table 1 shows the influence for different values of λ,$\;F_{r}$, and α on skin friction coefficients $\left(-Re_{x}^{1/2}C_{fx}\;\right)$ and $\left(-Re_{x}^{1/2}C_{fy}\right)$ when $R_{d}=\gamma =S=0$. Table 2 presents the numerical calculations of the Nusselt number $\left(Re_{x}^{-1/2}Nu_{x}\right)$ for numerous values of $F_{r}$, α, γ, $N_{t}$, $N_{b}$, Pr, and Sc. When $R_{d}=\gamma =S=0$, Table 1 and Table 2 show the findings with the results reported in [28]. We have observed an excellent agreement with these results. This demonstrates the validity and accuracy of the numerical technique used in this study for the present results. Among the most significant results of this research is that from the quantitative analysis in Table 1 and Table 2: increasing values of  λ,$\;F_{r}$, and α increase the drag forces in both directions, whilst an enhancement in γ values boosts the rate of heat transfer.

## 5. Conclusions

This article explores the rheological behavior flow of a nanofluid past a porous stretching sheet, with the study of heat generation or absorption and heat transfer. The Darcy–Forchheimer scheme of a porous medium was exhibited in the system of a flow problem. The higher-order chemical reaction and thermal non-linear radiation were taken into account. The numerical computations were executed for the non-linear system by utilizing the RKF45 technique with the shooting procedure. The findings are listed as follows:

- The velocity profile $f^{\prime }\left(\zeta \right)$ dwindled with augmented values of $M,\;F_{r},\;\lambda$, and α by keeping all the other parameters fixed in a single plot.
- The velocity profile $g^{\prime }\left(\zeta \right)$ was decreased with increasing values of M, $F_{r}$, and λ while it is increased with an increase in α.
- Increasing $F_{r}$, λ, $R_{d}$, $N_{t}$, S, and Bi values has always been reasoned to raise the temperature profile; nevertheless, in contrast, an upsurge in the parameters of Pr and α caused a reduction in the temperature.
- An increase in E and n increased the distributions of concentration. The opposite is true for increasing values of γ and δ.
- Decreasing $F_{r}$, λ, $R_{d}$, S, $N_{t}$ and Bi increased the distributions of concentration, while the opposite was true for the higher values of ζ.
- An increase in Pr, $N_{b}$ and Sc increased the distributions of concentration, while the opposite was true for the higher values of ζ.

## Figures and Tables

**Table 1:** Numerical values of skin friction coefficients for different values of parameters.

λ	$F_{r}$	α	Ref. [28]		Present Results	
			$-Re_{x}^{1/2}C_{fx}$	$-Re_{x}^{1/2}C_{fy}$	$-Re_{x}^{1/2}C_{fx}$	$-Re_{x}^{1/2}C_{fy}$
0.0	0.1	0.2	1.06945	1.67684	1.069452	1.676841
0.1			1.11471	1.81669	1.114711	1.816692
0.2			1.15830	1.94722	1.158301	1.947221
0.2	0.0		1.13041	1.19341	1.130412	1.193413
	0.1		1.15830	1.94722	1.158301	1.947223
	0.2		1.18561	1.96037	1.185611	1.960371
0.2	0.1	0.1	1.14160	2.54234	1.141603	2.542342
		0.3	1.17449	1.70234	1.174493	1.702343
		0.5	1.20663	1.47621	1.206632	1.476211

**Table 2:** Computational values of Nusselt number for diverse parameter values.

λ	$F_{r}$	α	γ	$N_{t}$	$N_{b}$	Pr	Sc	Ref. [28]	Present Results
								$Re_{x}^{-1/2}Nu_{x}$	$Re_{x}^{-1/2}Nu_{x}$
0.0	0.1	0.2	0.3	0.2	0.5	1.0	1.0	0.20448	0.204481
0.2								0.20248	0.202481
0.5								0.19970	0.199702
0.2	0.0	0.2	0.3	0.2	0.5	1.0	1.0	0.20278	0.202782
	0.2							0.20220	0.202203
	0.4							0.20164	0.201644
0.2	0.1	0.0	0.3	0.2	0.5	1.0	1.0	0.19458	0.194583
		0.3						0.20560	0.205601
		0.5						0.21080	0.210802
0.2	0.1	0.2	0.2	0.2	0.5	1.0	1.0	0.15148	0.151482
			0.5					0.27696	0.276963
			1.0					0.38194	0.381939
0.2	0.1	0.2	0.3	0.0	0.5	1.0	1.0	0.20306	0.203058
				0.5				0.20159	0.201591
				1.0				0.20004	0.200038
0.2	0.1	0.2	0.3	0.2	0.5	1.0	1.0	0.20248	0.202481
					1.0			0.20248	0.202481
					1.5			0.20248	0.202482
0.2	0.1	0.2	0.3	0.2	0.5	0.5	1.0	0.16685	0.166854
						1.0		0.20248	0.202482
						1.5		0.21949	0.219493
						1.0	0.5	0.20271	0.202712
							1.0	0.20248	0.202483
							1.5	0.20234	0.202344

## References

[1] Choi S.U.S., Eastman J.A. (1995). Enhancing Thermal Conductivity of Fluids with Nanoparticles.

[2] Prabhat N., Buongiorno J., Hu L.-W. (2012). Convective heat transfer enhancement in nanofluids: Real anomaly or analysis artifact?. J. Nanofluids.

[3] Hady F.M., Ibrahim F.S., Abdel-Gaied S.M., Eid M.R. (2012). Radiation effect on viscous flow of a nanofluid and heat transfer over a nonlinearly stretching sheet. Nanoscale Res. Lett..

[4] Alotaibi H., Althubiti S., Eid M.R., Mahny K.L. (2021). Numerical treatment of MHD flow of Casson nanofluid via convectively heated non-linear extending surface with viscous dissipation and suction/injection effects. Comput. Mater. Contin..

[5] Alotaibi H., Rafique K. (2021). Numerical Analysis of Micro-Rotation Effect on Nanofluid Flow for Vertical Riga Plate. Crystals.

[6] Eid M.R. (2016). Chemical reaction effect on MHD boundary-layer flow of two-phase nanofluid model over an exponentially stretching sheet with a heat generation. J. Mol. Liq..

[7] Eid M.R. (2017). Time-dependent flow of water-NPs over a stretching sheet in a saturated porous medium in the stagnation-point region in the presence of chemical reaction. J. Nanofluids.

[8] Boumaiza N., Kezzar M., Eid M.R., Tabet I. (2019). On numerical and analytical solutions for mixed convection Falkner-Skan flow of nanofluids with variable thermal conductivity. Waves Random Complex Media.

[9] Eid M.R., Al-Hossainy A.F., Zoromba M.S. (2019). FEM for blood-based SWCNTs flow through a circular cylinder in a porous medium with electromagnetic radiation. Commun. Theor. Phys..

[10] Eid M.R. (2020). Effects of NP shapes on non-Newtonian bio-nanofluid flow in suction/blowing process with convective condition: Sisko model. J. Non-Equili. Thermodyn..

[11] Lahmar S., Kezzar M., Eid M.R., Sari M.R. (2020). Heat transfer of squeezing unsteady nanofluid flow under the effects of an inclined magnetic field and variable thermal conductivity. Phys. A Statist. Mech. Appl..

[12] Alaidrous A.A., Eid M.R. (2020). 3-D electromagnetic radiative non-Newtonian nanofluid flow with Joule heating and higher-order reactions in porous materials. Sci. Rep..

[13] Eid M.R., Al-Hossainy A.F. (2020). Synthesis, DFT calculations, and heat transfer performance large-surface TiO_2_: Ethylene glycol nanofluid and coolant applications. Eur. Phys. J. Plus.

[14] Al-Hossainy A.F., Eid M.R. (2020). Structure, DFT calculations and heat transfer enhancement in [ZnO/PG+H2O]C hybrid nanofluid flow as a potential solar cell coolant application in a double-tube. J. Mater. Sci. Mater. Electron..

[15] Upreti H., Pandey A.K., Kumar M. (2020). Thermophoresis and suction/injection roles on free convective MHD flow of Ag–kerosene oil nanofluid. J. Comput. Des. Eng..

[16] Upreti H., Joshi N., Pandey A.K., Rawat S.K. (2021). Numerical solution for Sisko nanofluid flow through stretching surface in a Darcy–Forchheimer porous medium with thermal radiation. Heat Transf..

[17] Abbas Z., Hayat T. (2008). Radiation effects on MHD flow in a porous space. Int. J. Heat Mass Transf..

[18] Yazdi M.H., Abdullah S., Hashim I., Sopian K. (2011). Slip MHD liquid flow and heat transfer over non-linear permeable stretching surface with chemical reaction. Int. J. Heat Mass Transf..

[19] Hayat T., Imtiaz M., Alsaedi A., Mansoor R. (2014). MHD flow of nanofluids over an exponentially stretching sheet in a porous medium with convective boundary conditions. Chin. Phys. B.

[20] Ferdows M., Khan M., Alam M., Sun S. (2012). MHD mixed convective boundary layer flow of a nanofluid through a porous medium due to an exponentially stretching sheet. Math. Probl. Eng..

[21] Makinde O.D., Khan W.A., Culham J.R. (2016). MHD variable viscosity reacting flow over a convectively heated plate in a porous medium with thermophoresis and radiative heat transfer. Int. J. Heat Mass Transf..

[22] Eid M.R., Mahny K.L. (2018). Flow and heat transfer in a porous medium saturated with a Sisko nanofluid over a non-linearly stretching sheet with heat generation/absorption. Heat Transf. –Asian Res..

[23] Eid M.R., Mahny K.L., Muhammad T., Sheikholeslami M. (2018). Numerical treatment for Carreau nanofluid flow over a porous nonlinear stretching surface. Results Phys..

[24] Eid M.R., Mahny K.L. (2018). Unsteady MHD heat and mass transfer of a non-Newtonian nanofluid flow of a two-phase model over a permeable stretching wall with heat generation/absorption. Adv. Powder Technol..

[25] Eid M.R., Mahny K.L., Dar A., Muhammad T. (2020). Numerical study for Carreau nanofluid flow over a convectively heated nonlinear stretching surface with chemically reactive species. Phys. A Statist. Mech. Appl..

[26] Darcy H.P.G. (1856). Les Fontaines Publiques de la Ville de Dijon: Exposition et Application des Principes à Suivre et des Formules à Employer dans les Questions de Distribution d’eau, etc.

[27] Forchheimer P. (1901). Wasserbewegung durch Boden. Z. Ver. Dtsch. Ing..

[28] Muhammad T., Alsaedi A., Hayat T., Shehzad S.A. (2017). A revised model for Darcy–Forchheimer three-dimensional flow of nanofluid subject to convective boundary condition. Results Phys..

[29] Hayat T., Aziz A., Muhammad T., Alsaedi A. (2017). Darcy–Forchheimer three-dimensional flow of Williamson nanofluid over a convectively heated nonlinear stretching surface. Commun. Theor. Phys..

[30] Rasool G., Zhang T., Chamkha A.J., Shafiq A., Tlili I., Shahzadi G. (2020). Entropy generation and consequences of binary chemical reaction on MHD Darcy–Forchheimer Williamson nanofluid flow over non-Linearly Stretching Surface. Entropy.

[31] Rasool G., Shafiq A., Baleanu D. (2020). Consequences of Soret–Dufour effects, thermal radiation, and binary chemical reaction on Darcy Forchheimer flow of nanofluids. Symmetry.

[32] Ullah M.Z., Alshomrani A.S., Alghamdi M. (2020). Significance of Arrhenius activation energy in Darcy–Forchheimer 3D rotating flow of nanofluid with radiative heat transfer. Phys. A.

[33] Khan M.I., Alzahrani F., Hobiny A., Ali Z. (2020). Fully developed second order velocity slip Darcy-Forchheimer flow by a variable thicked surface of disk with entropy generation. Int. Commun. Heat Mass Transf..

[34] Upreti H., Pandey A.K., Kumar M., Makinde O. (2020). Ohmic heating and non-uniform heat source/sink roles on 3D Darcy–Forchheimer flow of CNTs nanofluids over a stretching surface. Arab. J. Sci. Eng..

[35] Eid M.R., Mabood F. (2020). Entropy analysis of a hydromagnetic micropolar dusty carbon NTs-kerosene nanofluid with heat generation: Darcy–Forchheimer scheme. J. Therm. Anal. Calorim..

[36] Muhammad R., Khan M.I., Jameel M., Khan N.B. (2020). Fully developed Darcy-Forchheimer mixed convective flow over a curved surface with activation energy and entropy generation. Comput. Meth. Prog. Biomed..

[37] Eid M.R., Mabood F. (2020). Two-phase permeable non-Newtonian cross-nanomaterial flow with Arrhenius energy and entropy generation: Darcy-Forchheimer model. Phys. Scr..

[38] Riasat S., Ramzan M., Kadry S., Chu Y.-M. (2020). Significance of magnetic Reynolds number in a three-dimensional squeezing Darcy–Forchheimer hydromagnetic nanofluid thin-film flow between two rotating disks. Sci. Rep..

[39] Hayat T., Khan S.A., Alsaedi A., Fardoun H.M. (2020). Heat transportation in electro-magnetohydrodynamic flow of Darcy-Forchheimer viscous fluid with irreversibility analysis. Phys. Scr..

[40] Singh K., Pandey A.K., Kumar M. (2021). Numerical approach for chemical reaction and suction/injection impacts on magnetic micropolar fluid flow through porous wedge with Hall and ion-slip using Keller Box method. Waves Random Complex Media.

